# Effectiveness and Safety of OnabotulinumtoxinA in Adolescent Patients with Chronic Migraine

**DOI:** 10.3390/toxins16050221

**Published:** 2024-05-11

**Authors:** Laura Gómez-Dabó, Edoardo Caronna, Rut Mas-de-les-Valls, Víctor J. Gallardo, Alicia Alpuente, Marta Torres-Ferrus, Patricia Pozo-Rosich

**Affiliations:** 1 Headache Clinic, Neurology Department, Vall d’Hebron Hospital, Universitat Autònoma de Barcelona, 08035 Barcelona, Spain; laura.gomezdabo@vallhebron.cat (L.G.-D.); edoardo.caronna@vhir.org (E.C.); alicia.alpuente@vallhebron.cat (A.A.); marta.torres@vallhebron.cat (M.T.-F.); 2 Headache and Neurological Pain Research Group, Vall d’Hebron Institut de Recerca (VHIR), Department of Medicine, Universitat Autònoma de Barcelona, 08035 Barcelona, Spain; rut.mas_de_les_valls@vhir.org (R.M.-d.-l.-V.); victor.gallardo@vhir.org (V.J.G.)

**Keywords:** migraine, onabotulinumtoxinA, adolescents, teenagers, children

## Abstract

Chronic migraine (CM) significantly affects underage individuals. The study objectives are (1) to analyze the effectiveness and safety of onabotulinumtoxinA (BTX-A) in adolescents with CM; (2) to review the literature on BTX-A use in the pediatric population. This prospective observational study included patients under 18 years old with CM treated with BTX-A (PREEMPT protocol) as compassionate use. Demographic, efficacy (monthly headache days—MHD; monthly migraine days—MMD; acute medication days/month—AMDM) and side effect data were collected. A ≥ 50% reduction in MHD was considered as a response. Effectiveness and safety were analyzed at 6 and 12 months. A systematic review of the use of BTX-A in children/adolescents was conducted in July 2023. In total, 20 patients were included (median age 15 years [14.75–17], 70% (14/20) females). The median basal frequencies were 28.8 [20–28] MHD, 18 [10–28] MMD and 10 [7.5–21.2] AMDM. Compared with baseline, at 6 months (*n* = 20), 11 patients (55%) were responders, with a median reduction in MHD of −20 days/month (*p* = 0.001). At 12 months (*n* = 14), eight patients (57.1%) were responders, with a median reduction in MHD of −17.5 days/month (*p* = 0.002). No adverse effects were reported. The literature search showed similar results. Our data supports the concept that BTX-A is effective, well tolerated, and safe in adolescents with CM resistant to oral preventatives.

## 1. Introduction

Migraine is a common and disabling neurological disorder that affects all ages. In children and adolescents, the prevalence of migraine is estimated to be around 11% [1], increasing from childhood to adolescence—from 5% among children under 10 years old to 15% among teenagers [2]—with an incidence peak occurring around the initiation of puberty, typically at the age of 13 [3]. Concerning chronic migraine (CM), it is reported to affect from 0.2% to 12% of children/adolescents [1]. Often, anxiety, depression, or other psychological factors are associated with it, and it is not uncommon to encounter young patients with daily headache [4,5,6,7]. In addition, according to the Global Burden of Disease study, headache is ranked as the second most disabling disease worldwide among individuals between 10 and 24 years old [8]. 

While the overall burden of migraine is acknowledged to be substantial in the overall population [8], children/adolescents have to face further major issues: (1) no preventive treatment is specifically approved in this age group for migraine, whose pharmacological management is therefore only based on recommendations and is therapeutically limited [9]; (2) inadequate disease management during early stages affects social and educational domains with a severe impact later on in life (e.g., career development) [10]. 

OnabotulinumtoxinA (BTX-A) has demonstrated its efficacy and safety in randomized controlled trials (PREEMPT trials 1, 2) [11,12] and in the real world for CM prevention in adults [13,14,15] and could potentially be a therapeutic tool for children/adolescents, especially for cases that are more treatment resistant, yet data on its use in this specific population are scarce [16,17]. 

Thus, we aimed to (1) analyze the effectiveness and safety profile of BTX-A in our real-world cohort of underage migraine patients and (2) review the current evidence on the use of BTX-A in children/adolescents with CM. 

## 2. Results

### 2.1. Case Series 

#### 2.1.1. Patient Characteristics 

A total of 20 patients met the inclusion criteria. Of those, 14 patients were female (70%), with a median age of 15 years old [IQR 14.75–17]. Relevant comorbidities included obesity (1/20; 5%), anxiety (2/20; 10%) and depression (3/20; 15%). Patients had a median age of migraine onset of 12 years [IQR 8–14] and a median age of migraine chronification of 14 [IQR 12.5–15.5]. At baseline, the median monthly headache days (MHD) was 28 days/month [IQR 20–28], the median monthly migraine days (MMD) was 18 days/month [IQR 10–28] and the median of acute medications days/month (AMDM) was 10 days/month [IQR 7.5–21.2]. The median number of failed previous preventive treatments was one, with 20% of patients (4/20) with stable concomitant oral preventive medications. All baseline characteristics are shown in Table 1. 

#### 2.1.2. Effectiveness

At 6 months (after 2 BTX-A doses), the median reduction in MHD was −20 days/month (M0 median 28 days/month [IQR 20–28], M6 median 8 days/month [5.5–26.5] (*p* = 0.001)), the median reduction in MMD was −13.5 days/month (M0 median 18 days/month [10.0–28.0], M6 median 4.5 days/month [2.8–7.0] (*p* < 0.001)), and in AMDM, it was −6 days/month (M0 median 10 days/month [7.5–21.2], M0 median 4 days/month [2.5–7.5] (*p* = 0.01)). Eleven out of twenty patients were responders (55%). 

**Figure 1:**
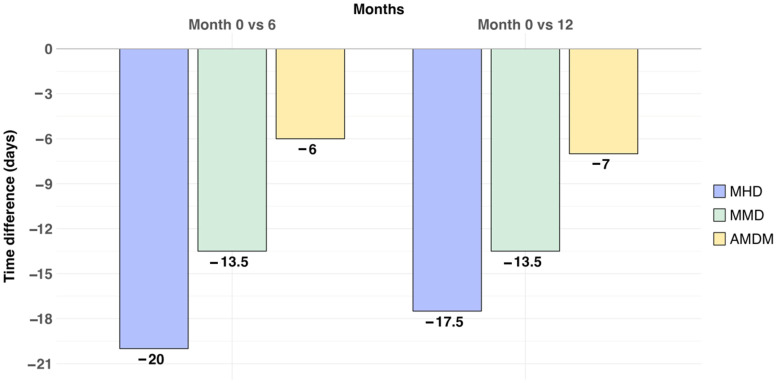
Median reduction in headache frequencies across the study period (baseline 0–month, 6–month and 12–month follow-up). The median reduction in MHD, MMD and AMDM is represented in days/month. MHD: monthly headache days; MMD: monthly migraine days; AMDM: acute medication days/month.

At 12 months, there was a significant reduction in MHD (−17.5 days/month, M12 median 10.5 days/month [5.2–21.5]; *p* = 0.002), MMD (−13.5 days/month, M12 median 4.5 [2.8–7.8] *p* = 0.014) and in AMDM (−7 days/month; M12 median 3 days/month [2.0–6.0]; *p* = 0.028). Eight out of fourteen patients were responders (57.1%). Figure 1 shows changes in headache frequencies across the study period. 

Furthermore, although in our study no direct comparison between 6 months and 12 months of treatment has been performed, the results show a tendency toward a maintained BTX-A response. 

#### 2.1.3. Safety 

BTX-A treatment was well tolerated, with no minor or major adverse events reported. None of the patients discontinued treatment due to side effects. 

### 2.2. Narrative Review

**Figure 2:**
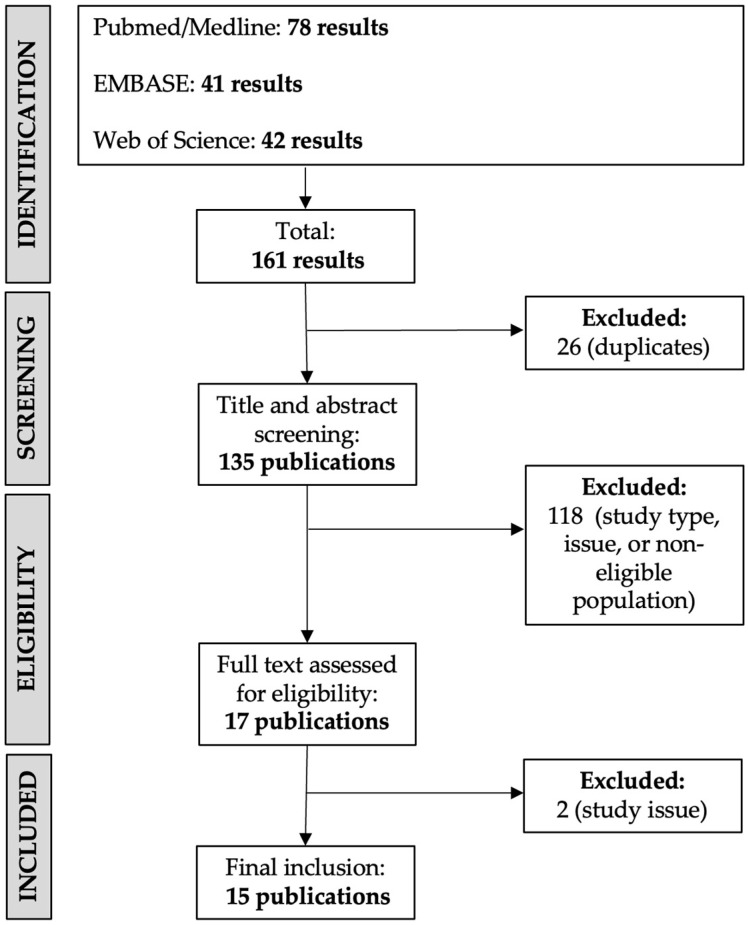
Flowchart of study selection for review.

The PRISMA guideline-based search process is summarized in Figure 2. A total of 160 results were screened, and a subset of 15 publications were ultimately included. Published studies have focused on the efficacy of BTX-A treatment as well as on describing treatment safety and tolerability (Table 2). 

Since the first published studies in 2009, only two randomized control trials (RCTs) have been published. The first one [16] was published in January 2020 and consisted of a single-treatment, multicenter, double-blind study. Treatment with BTX-A was 75 U or 155 U in 31 injection sites following the PREEMPT protocol. The three treatment arms (placebo-PBO, 75 U and 155 U BTX-A) reduced headache frequency days and severe headache days, but without significant differences between them. However, the study had two important limitations: first, there was a probable higher PBO effect than expected because of a 2:1 chance of patients receiving active treatment; and second, efficacy was evaluated after a single-dose treatment. 

The second RCT [17] was published in April 2020 and used an AB|BA crossover design with two phases: two sets of treatments in a double-blind period and two additional sets of treatments in which patients could elect between BTX-A or standard care. Fifteen patients were randomized, and treatment consisted of 155 U of BTX-A in 31 injection sites following the PREEMPT protocol. Compared with patients who received PBO (6/15), 9/15 of the patients who received BTX-A presented a statistically significant reduction in headache frequency (median frequency in days of 20 ([QR 7 to 17] vs. 28 [IQR 23 to 28]; *p* = 0.038), intensity (median in intensity in a 0 to 10 pain numeric rating scale of 5 [IQR 3 to 7] vs. 7 [IQR 5 to 9]; *p* = 0.047) and in disability assessed with PedMIDAS score (median grade of 3 [IQR 2 to 4] vs. 4 [IQR 4 to 4]; *p* = 0.047). Interestingly, two patients who were non-responders to BTX-A were diagnosed with idiopathic intracranial hypertension, and the re-run analysis without them showed greater differences between both interventions. 

Regarding the efficacy of BTX-A, with respect to headache frequency, all studies showed a reduction in MHD, yielding proportions of treatment responders (i.e., ≥50% reduction in MHD) ranging from 45% to 70% [18,19]. In terms of headache intensity, often measured with the Visual Analogue Scale (VAS), a significant reduction was commonly associated with BTX-A across a substantial proportion of studies, yielding a mean VAS reduction from 1.28 to 5.2 points [17,19,20,21,22,23,24]. Concerning the duration of headache attack, there are a few controversial results regarding its improvement, with two studies favoring positive effects [17,23] and one showing no effect [16]. Moreover, one study suggests that treatment with BTX-A might contribute to reducing the occurrence of auras [25]. 

Furthermore, regarding the safety of BTX-A, no severe adverse events have been documented, except from a case report describing reversible, progressively decreasing pulmonary function in a 15-year-old female treated with 150 U of BTX-A. It is noteworthy that the patient had pre-existing relevant health problems consisting of a common variable immunodeficiency undergoing routine IVIG infusions and non-steroidal hypersensitivity [26]. Most of the reported side effects were mild, including injection-site erythema, edema and/or pain, pruritus, headache, eyebrow elevation, neck pain and/or weakness, dizziness and nausea, which were not described as reasons for discontinuation in any of the included studies. 

Moreover, some studies have investigated the potential impact of comorbidity on the response to BTX-A, with controversial results. Goenka et al. [27] showed that anxiety (GAD-7 score greater than 15) was significantly higher among non-responders (67%) compared to responders (42%) (*p* = 0.040). However, Papetti et al. [18] observed no impact of anxiety (GAD-7) or depression (PHQ-9) on the BTX-A response. Additionally, a limited number of studies have investigated how BTX-A might enhance the quality of life in pediatric patients using the PedMIDAS score, also with conflicting outcomes; two studies support a significant improvement in disability [17,27] but another does not [16]. 

## 3. Discussion

Consistent with previous research, our study supports the concept that BTX-A can be an effective and safe treatment for the adolescent population with CM. 

Considering headache effectiveness, nearly 60% of patients were responders at 12 months of follow-up, aligning with findings from prior investigations [16,17,18,19,21,22,30]. Both MHD and MMD were significantly reduced following treatment initiation, with a median reduction of 8 and 4.5 days per month, respectively. Furthermore, treatment with BTX-A was accompanied by a reduction in the utilization of acute headache medications, potentially mitigating the risk of medication overuse headache [31]. 

In addition, our study results also underscore the favorable adherence to BTX-A treatment by patients. BTX-A treatment was well tolerated, with no major adverse events observed, similar to the published studies (as detailed in our narrative review results). 

Our narrative review discloses that evidence is scarce because of multiple limitations. First, there are very few studies meeting our review’s inclusion criteria (15 studies over a span of 15 years). Secondly, the quality of the studies is generally low, with only two RCTs [16,17] and most of them being retrospective case series studies. 

In addition, the patient sample sizes in these studies are small, restricting the ability to draw statistically significant conclusions. Furthermore, studies use different BTX-A doses, and those conducted prior to the publication of the PREEMPT protocol did not adhere to established injection sites, making precise inter-study comparisons difficult. 

Lastly, in most of the studies, the concurrent use of other preventive medications was allowed but with unclear descriptions of the types and dosages of and changes in oral medications, potentially representing confounding factors. 

Concerning the limitations of our clinical study, we are aware that, like previous studies, our sample size is small; however, this is a well-phenotyped cohort relying on robust data (e.g., headache e-diaries). Also, we did not have assessed Patient Reported Outcomes Measures such as PedMIDAS, which would have been interesting to further explore patient perception and the impact on patient quality of life. Finally, no control group was included in the study. We cannot exclude, for this reason, a placebo effect, which is known to be high in the pediatric population; nevertheless, given the 12-month duration of the study and the sustained response to BTX-A, the influence of this effect may be limited.

With all this, it is essential to undertake more studies, including RCTs, to bolster the evidence supporting the use of BTX-A in adolescents. 

## 4. Conclusions

Both the literature systematic review and our own prospective study show that BTX-A stands as an effective and safe therapeutic option for adolescents with chronic migraine. However, to firmly establish BTX-A as a standard treatment, there remains a requirement for additional high-quality empirical substantiation. Properly treating migraine in these stages of life can help teenagers perform in their academic and personal life and can control the progression of the disease in order to start adulthood with a controlled migraine disease. 

## 5. Materials and Methods

### 5.1. Study Design, Population, and Outcomes 

This is a prospective pilot study conducted in the Headache Clinic of a tertiary hospital in Spain. We included all consecutive outpatients attended between October 2019 and May 2023, meeting the following inclusion criteria: (1) age < 18 years old; (2) diagnosis of CM according to the International Classification of Headache Disorders-3rd Edition (ICHD-3) [32]; (3) failure with one or more migraine oral preventive treatment (including contraindication); (4) treatment with BTX-A 195 U every 3 months, according to the PREEMPT protocol [28], on compassionate use. 

BTX-A was offered as compassionate use after more than 1 failure with oral preventive treatment, considering the absence of an approved oral preventive treatment for CM in the underage population, and upon patients’ and legal tutors’ approvals. 

Concomitant oral preventive treatments were allowed, provided that their doses were stable during the study period (at least 1 month before starting BTX-A), in patients who wished to continue treatment despite limited efficacy (defined as a reduction ≤ 50% in MHD). We included patients with daily headache. 

BTX-A was administered to all patients according to the PREEMPT injection paradigm [30], using a total dose of 195 units (U) injected in 39 sites (frontalis 20 U in 4 sites; corrugator 10 U in 2 sites; procerus 5 U in 1 site; occipitalis 40 U in 8 sites; temporalis 50 U in 10 sites; trapezius 50 U in 10 sites; cervical paraspinal muscle group 20 U in 4 sites) every 12 weeks. 

We collected demographic data (age, sex) and comorbidities (including anxiety, depression, obesity, cardiovascular disease, neurovascular disease). Additional data included migraine onset and age of diagnosis (years), the presence of aura and other migraine characteristics. Through electronic headache diaries (e-diaries) we collected MHD, MMD and AMDM values. We evaluated presence of any adverse event. At least three visits were stablished, including baseline (M0), 6-month (M6) and 12-month (M12) follow-ups. 

The primary outcome of this study was to assess the effectiveness of BTX-A in terms of reduction in MHD at 6 months. Secondary outcomes were reduction in MHD at 12 months, reduction in MMD and AMDM at 6 and 12 months and the proportion of responders at 6 and 12 months. We defined response to BTX-A as ≥50% reduction in MHD. We evaluated BTX-A safety, reason for discontinuation and retention rate. 

### 5.2. Statistical Analysis 

The statistical analysis was conducted using R version 4.3.1 (2023-06-16). 

Sex, aura and all comorbidities were processed as categorical variables described with frequency and percentage, while age, evolution, MHD, MMD and AMDM were processed as quantitative variables described with median and quantiles.

According to the Shapiro test results assessing the normality of data, a paired t-test (normally distributed data) or Wilcoxon test (not-normally distributed data) was used to study differences in frequencies and medication at months 0–6 and 0–12. There was no need to adjust the *p*-value, as this was an exploratory study. 

A statistical power calculation prior to the study was not conducted because the sample size was based on the available data. No missing values were obtained. We considered two-tailed test *p* values < 0.05 statistically significant. 

### 5.3. Narrative Review: Search Strategy and Study Selection

A literature search was conducted in July 2023 using the following electronic databases: PubMed/MEDLINE, EMBASE and Web of Science. Separate searches were performed within each database (the search term strategy is described in Supplementary Material S1). Eligibility criteria were established before the literature search was conducted. Studies involving patients under the age of 18 years and in English were included. Reviews, expert opinions, practice guidelines, book chapters and abstracts or congress posters were also excluded. 

Using the aforementioned criteria, E.C. and L.G.-D. independently reviewed the titles and abstracts of all resulting publications. Studies that met the exclusion criteria and duplicates were removed. Full texts of the remaining papers were then analyzed. For the risk of bias assessment, Cochrane Collaboration’s tool was used for randomized clinical trials (RCTs) [33] and the Risk of Bias in Non-randomized Studies of Exposures (ROBINS-E Version 20) was used for observational epidemiologic studies [34].

## Figures and Tables

**Table 1:** Basal characteristics and demographics.

Baseline Characteristics	
Age, years, median [IQR]	15 [14.75, 17]
Gender, female, n (%)	14 (70%)
Migraine onset age, median [IQR]	12 [8, 14]
Migraine diagnosis age, median [IQR]	15 [14, 16]
Migraine age of chronification, median [IQR]	14 [12.5–15.5]
Aura, n (%)	1 (5%)
Hypertension, n (%)	0 (0%)
Obesity, n (%)	1 (5%)
Anxiety, n (%)	2 (10%)
Depression, n (%)	3 (15%)
Cardiovascular disease, n (%)	0 (0%)
Neurovascular disease, n (%)	0 (0%)
MHD, days/month, median [IQR]	28 [20, 28]
MMD, days/month, median [IQR]	18 [10, 28]
AMDM, days/month, median [IQR]	10 [7.5, 21.25]
Failed preventive treatments, n (%)	19 (95%)
Beta-blockers	5 (25%)
Antidepressants	15 (75%)
Neuromodulators	5 (25%)
Calcium channel blockers	9 (45%)
Angiotensin receptor blockers	1 (5%)
Number of treatments failed, n (%)	
1 class	9 (45%)
2 classes	5 (25%)
3 classes	3 (15%)
4 classes	2 (10%)
Other ongoing treatments, n (%)	4 (20%)
Beta-blockers	1 (5%)
Antidepressants	3 (15%)
Neuromodulators	0 (0%)
Calcium channel blockers	0 (0%)
Angiotensin receptor blockers	0 (0%)

Six patients were not analyzed at 12 months: three patients had not reached the 12 months when the analysis was conducted, one patient went from adolescence to adulthood before month 12, one patient discontinued because of clinical improvement and one patient discontinued because of partial response to BTX-A treatment. AMDM: acute medication days/month; MHD: headache days/month; MMD: monthly migraine days.

**Table 2:** Narrative review of the use of BTX-A in children and adolescents with migraine.

Ref	Type of Study	Study Participants	Primary Outcome	Result	Risk of Bias
[16]	RCT of one dose of BTX-A (155 U or 74 U) versus PBO	N = 125 randomized patients with CM, 115/125 (92%) completed the study Age 15.1 (±1.5 years) Female 98/125 (78.4%) Follow-up: 3 months	Change in frequency of headache days from baseline at week 12	All treatments reduced frequency with no significant differences between them: −6.3 (−8.5, −4.2) in BTX-A 155 U; −6.4 (−8.8, −4.0) in BTX-A 75 U; PBO −6.8 (−9.6, −4.1) in PBO; *p* ≥ 0.474.	Selection bias: low Performance bias: low Detection bias: low Attrition bias: low Reporting bias: low Overall risk of bias: low
[17]	2-phase RCT (double-blind and open-label) of 155 U BTX-A (PREEMPT protocol) versus PBO	N = 15 patients with CM. 11/15 received three BTX-A treatmentsAge 15 (±1 year) Female 13/15 (87%) Follow-up: 6 weeks	To evaluate efficacy of BTX-A (reduction in frequency, intensity, duration, and disability) in patients with CM versus PBO.	Reduction in frequency (20 (7 to 17) vs. 28 (23 to 28); *p* = 0.038), intensity (5 (3 to 7) vs. 7 (5 to 9); *p* = 0.047) and disability (PedMIDAS 3 (2 to 4) vs. 4 (4 to 4); *p* = 0.047). No significant headache duration difference (10 (2 to 24) vs. 24 (4 to 24); *p* = 0.148).	Selection bias: unclear risk Performance bias: low Detection bias: low Attrition bias: low Reporting bias: low Overall risk of bias: low
[18]	Retrospective study of prospective case series of CM treated with BTX-A 155–195 U (PREEMPT protocol)	N = 46 CM patients (analysis performed on 43/46) Age 14.7 (±1.5 years) Female 37/46 (80.4%) Follow-up: 17.6 ± 13.7 months	Change in MHD from BL during 3 cycles of BTX-A	68% had a significant reduction in the frequency of MHD within the 3 cycles, and 45% were responders (reduction ≥ 50% in MHD).	Due to confounding: high Measurement of the exposure: low In selection: high In post-exposure interventions: low Due to missing data: low In measurements of outcomes: low In selection of the reported result: low Overall risk of bias: high
[19]	Retrospective and prospective case series of CM treated with 155 U BTX-A (PREEMPT protocol)	N = 56 CM patients (analysis performed on 34/56) Age 17.5 (±2 years). 3/34 patients from 19–29 years Female 30/34 (88.2%) Follow-up: 9 months	To analyze the efficacy of BTX-A (reduction in headache frequency ≥ 50% and intensity) and reasons for discontinuation	73% of responders (25/34 ≥ 50% reduction in frequency). A reduction in intensity with each additional BTX-A dose was observed (BL VAS 8.3 ± 0.8 vs. 4th dose 3.4 ± 1.4; *p* < 0.001). Discontinuation in 22/34 (11/22 moving out of the area, 3/22 minor side-effects and 8/22 non-response).	Due to confounding: high Measurement of the exposure: low In selection: high In post-exposure interventions: SC Due to missing data: SC In measurements of outcomes: low In selection of the reported result: low Overall risk of bias: high
[20]	Retrospective chart review on patients with CM with ≥ 2 BTX-A administrations (doses not specified)	N = 32 patients with CM Age 16.09 (±1 year) Female 30/32 (93.8%) Follow-up: not clearly specified	Comparison of headache frequency, intensity and duration across BL, first and second BTX-A Inj	Reduction in frequency (mean reduction of 6.5 days between BL and Inj1 (*p* < 0.01); 7 days between BL and Inj2 (*p* <0.01)) and intensity (BL 6.8 ± 1.51; Inj1 6.17 ± 2.12; Inj2 5.52 ± 6.2; *p* = 0.003). No significant reduction in duration.	Due to confounding: high Measurement of the exposure: high In selection: high In post-exposure interventions: low Due to missing data: SC In measurements of outcomes: low In selection of the reported result: SC Overall risk of bias: high
[21]	Retrospective case series of patients with CDH with “StiBo” approach ^+^. Mean total BTX-A dose 32.7 U (20–90 U).	N = 5 patients (1 lost follow-up) Age 13 (±2.4 years) Female 4/5 (80%) Follow-up: 17 months	Long-term evaluation (10 year) of StiBo project regarding headache	Reduction in headache frequency from 20 (15–30) to 5 (0–15) and headache intensity (VAS) from 6.6 (6–8) to 2.4 (0–5).	Due to confounding: high Measurement of the exposure: low In selection: high In post-exposure interventions: SC Due to missing data: high In measurements of outcomes: high In selection of the reported: SC Overall risk of bias: high
[22]	Retrospective case series of patients with CM. Mean BTX 173.23 ± 35.03 U (PREEMPT protocol)	N = 65 patients with CM. Age 15 (11–18 years) Female % not known. Follow-up: 6 weeks	To analyze tolerability, side effects and pain scores (VAS) of BTX-A	2/65 patients presented mild side effects (1/2 not related to BTX-A). Significant reduction in pain score with an average pain score decrease of 5.2 ± 2.2 on VAS.	Due to confounding: high Measurement of the exposure: low In selection: high In post-exposure interventions: low Due to missing data: high In measurements of outcomes: SC In selection of the reported result: lowOverall risk of bias: high
[23]	Retrospective chart review of refractory CM treated with BTX-A 155–215 U (PREEMPT protocol)	N= 10 patients with refractory migraine ‡ Age 15.1 (±2.95 years) Female 7/10 (70%)Follow up: 2.5 years.	Decrease in frequency (MHD), duration and intensity of migraine episodes with BTX-A compared to pretreatment	Reduction in frequency (15 (8 to 29) vs. 4 (2 to 10); *p* < 0.0001), intensity (6 (4 to 8) vs. 4 (2 to 5); *p* = 0.0063) and duration in hours (8 (0 to 24) vs. 0.8 (0.3 to 7); *p* = 0.025).	Due to confounding: high Measurement of the exposure: low In selection: high In post-exposure interventions: low Due to missing data: high In measurements of outcomes: low In selection of the reported result: SC Overall risk of bias: high
[24]	Retrospective cohort of adolescents with CM treated with BTX-A 155 U–185 U (PREEMPT protocol)	N = 30 patients with CM Age 16.5 (±1.83 years) Female 24/30 (80%) Follow-up: 12–24 months	To analyze response in terms of intensity and frequency of BTX-A in children with CM	Significant reduction in headache intensity (VAS 7.47 ± 1.89 to 4.34 ± 3.02; *p* < 0.001) and frequency (24.4 ± 7.49 to 14.8 ± 12.52 MHD; *p* < 0.001).	Due to confounding: high Measurement of the exposure: low In selection: high In post-exposure interventions: low Due to missing data: low In measurements of outcomes: SC In selection of the reported: low Overall risk of bias: high
[25]	Retrospective case series of 11 patients with CM. Pediatric patient treated with BTX-A 155–200 U standard fixed-site protocol	N = 1/11 pediatric patient 16-year-old female with CM Follow-up: 1 year	Description of effectiveness of BTX-A in a patient with CM	Reduction in severe migraine headaches preceded by visual and motor aura (severe headaches reduced to 8 days per month, and severe motor aura from 3/week to 1/3 months).	Due to confounding: NI Measurement of the exposure: low In selection: high In post-exposure interventions: low Due to missing data: high In measurements of outcomes: high In selection of the reported result: low Overall risk of bias: high
[26]	Case report patient with CM; 150 U BTX-A	N = 1 patient 15 years old female with CM Follow-up: 24 months	Description of progressively decreasing pulmonary function during BTX-A administration	FVC from 109% to 55% over 24 months; Pulmonary function recovered immediately after discontinuation of BTX-A therapy.	Due to confounding: NI Measurement of the exposure: lLow In selection: H high In post-exposure interventions: lLow Due to missing data: hHigh In measurements of outcomes: Hhigh In selection of the reported result: SC Overall risk of bias: hHigh
[27]	Retrospective and prospective case series of CM treated with 155 U BTX-A (PREEMPT protocol)	N = 56 CM patients (analysis performed on 34/56). Age 17.5 (±2 years) Female 30/34 (88.2%) Follow-up: 9 months Same cohort (23) but different primary endpoint.	To evaluate the effectiveness of BTX-A and the impact of comorbidity (anxiety) on its response	GAD-7 score greater than 15 was significantly higher in non-responders (67%) vs. responders (42%); *p* = 0.040.	Due to confounding: high Measurement of the exposure: low In selection: high In post-exposure interventions: SC Due to missing data: SC In measurements of outcomes: low In selection of the reported result: low Overall risk of bias: high
[28]	Retrospective case series of CDH treated with BTX-A 100 U fixed Inj sites	N = 10 patients (5 with CM). Age 15.5 (SD not available). Female 7/10 (70%). Follow-up: not clearly specified	Description of tolerability and effectiveness of BTX-A in intractable pediatric patients with CDH	No serious AE, 3/10 minor events. 4/10 (40%) clinically meaningful reliefs of headache symptoms. 3/5 (60%) patients with CM were responders in terms of intensity (3/3 40–50% reduction) and/or frequency (2/3; 40–50% reduction); *p* not available	Due to confounding: high Measurement of the exposure: low In selection: high In post-exposure interventions: low Due to missing data: high In measurements of outcomes: high In selection of the reported result: SC Overall risk of bias: high
[29]	Qualitative review of patients with CM treated with 100 U BTX following a combination of fixed-site and follow-the-pain pattern (9–63 Inj sites)	N = 12 patients with CM (analysis performed on 6/10 with long-term BTX-A) Age 14.8–18.1 years Female 6/6 (100%) Follow-up: 3–29 months	Decrease in headache frequency, pain intensity and/or duration of headache. Evaluation of headache in QoL	6/6 had a reduction in migraine frequency days (variable) and 3/6 a decrease in intensity. 25% improvement in the total scores of MSQOL (although comparison scores prior to starting BTX and at 3 months failed to reach significance).	Due to confounding: high Measurement of the exposure: SC In selection: high In post-exposure interventions: SC Due to missing data: high In measurements of outcomes: high In selection of the reported result: SC Overall risk of bias: high

AE: adverse events; BL; baseline; BTX-A: onabotulinumtoxinA; CDH: chronic daily headache; CM: chronic migraine; FVC: forced vital capacity; MHD: headache days/month; Inj: injection; MSQOL: Migraine-Specific Quality-Of-Life instrument; QoL: quality of life; PBO: placebo; Ref: reference; RCT: randomized control trial; SC: some concerns; VAS: Visual Analogue Scale (from 0 to 10); vs: versus. ‡ Refractory migraine defined in the study as chronic migraine by IHS criteria and failure of neuropathic medication, abortive treatments, preventative medications or opioid therapy to provide significant relief. ^+^ StiBo approach: short-term integrative intervention including BTX-A.

## Data Availability

All data are available, and any anonymized data will be shared upon request from any qualified investigator.

## Supplementary Materials

The following supporting information can be downloaded at: https://www.mdpi.com/article/10.3390/toxins16050221/s1, Supplementary Material S1. Search term strategy.

## Abbreviations

AMDM	Acute medication days/month
BAI	Beck Anxiety Inventory Scale
BDI	Beck Depression Inventory
BTX-A	OnabotulinumtoxinA
CM	Chronic migraine
IQR	Interquartile range
ICHD-3	International Classification of Headache Disorders
MHD	Monthly headache days
MMD	Monthly migraine days
RCT	Randomized clinical trial
PBO	Placebo
U	Unit
