# Supplementary material for: Effectiveness and Safety of OnabotulinumtoxinA in Adolescent Patients with Chronic Migraine
Source: Toxins (Basel). 2024 May 11;16(5):221. doi: 10.3390/toxins16050221 (PMC11126010; doi:10.3390/toxins16050221)
Supplement: Supplementary file 1 [file toxins-16-00221-s001.zip › toxins-2973009-SI.pdf]

Supplementary Materials: Effectiveness and Safety of OnabotulinumtoxinA in Adolescent Patients with Chronic Migraine

Supplementary Material S1. Search term strategy.

Pubmed/MEDLINE ("Migraine Disorders" [Mesh]) OR (Migraine*[Title/Abstract])) AND (((("Botulinum Toxins, Type A" [Mesh]) OR (Botox[Title/Abstract])) OR ("Botulinum Toxin A"[Title/Abstract])) OR (OnabotulinumtoxinA[Title/Abstract])).

EMBASE ('migraine'/exp OR 'migraine*': ab,kw,ti) AND (botox:ti,kw,ab OR 'onabotulinumtoxina:ti,kw,ab) AAND ([newborn]/lim OR [infant]/lim OR [child]/lim OR [preschool]/lim OR [school]/lim OR [adolescent]/lim).

Web of Science: (('pediatr*') OR (adolescen*) OR (child*)) AND (('botulinum toxin a') OR (botox) OR ('botulinum toxin a') AND ('migraine*')
